# Sea level rise drives increased tidal flooding frequency at tide gauges along the U.S. East and Gulf Coasts: Projections for 2030 and 2045

**DOI:** 10.1371/journal.pone.0170949

**Published:** 2017-02-03

**Authors:** Kristina A. Dahl, Melanie F. Fitzpatrick, Erika Spanger-Siegfried

**Affiliations:** 1 Dahl Scientific, San Francisco, California, United States of America; 2 Independent Consultant, South Hobart, Tasmania, Australia; 3 Climate & Energy Program, Union of Concerned Scientists, Cambridge, Massachusetts, United States of America; Bristol University/Remote Sensing Solutions Inc., UNITED STATES

## Abstract

Tidal flooding is among the most tangible present-day effects of global sea level rise. Here, we utilize a set of NOAA tide gauges along the U.S. East and Gulf Coasts to evaluate the potential impact of future sea level rise on the frequency and severity of tidal flooding. Using the 2001–2015 time period as a baseline, we first determine how often tidal flooding currently occurs. Using localized sea level rise projections based on the Intermediate-Low, Intermediate-High, and Highest projections from the U.S. National Climate Assessment, we then determine the frequency and extent of such flooding at these locations for two near-term time horizons: 2030 and 2045. We show that increases in tidal flooding will be substantial and nearly universal at the 52 locations included in our analysis. Long before areas are permanently inundated, the steady creep of sea level rise will force many communities to grapple with chronic high tide flooding in the next 15 to 30 years.

## Introduction

Sea level rise has the potential to inundate significant stretches of the U.S. coastline by the end of this century [1]. With higher sea levels, local flooding thresholds can be reached more easily during average high tides. In the absence of coastal adaptation measures to protect against rising seas, some coastal areas could fall below the high tide line by the end of the century. Before that permanent inundation occurs, however, unprotected coastal areas could experience more frequent flooding with high tides.

Global sea level rose by an average of 1.2–1.7 mm per year over the course of the 20^th^ century [2,3]. The rise in sea level is accelerating both globally and regionally in many places. From 1993 to 2010, the global rate has accelerated to 3.0 +/- 0.7 mm per year [2–5]. This acceleration is attributed mainly to ocean warming, a quickening pace of land ice loss, and a net transfer of groundwater from the land into the sea [6–9].

Local sea level trends can differ from the global average due to factors including land subsidence, tectonics, changes in ocean circulation, gravitational or sea level fingerprinting, groundwater pumping, and dredging [6,10–13]. The stretch of coastline from Nova Scotia to the Gulf of Mexico faced some of the world’s fastest rates of sea level rise in the twentieth century—from 2.8 mm per year in Boston to 9.1 mm per year at Grand Isle, Louisiana [14]. The Northeast U.S., in particular, is a “hot spot” of rapid sea level rise, possibly caused by climate-induced weakening of the Atlantic Meridional Overturning Circulation (AMOC) and its upper branch, the Gulf Stream [11,15,16].

In this study, we focus on tidal flooding events because they are an increasingly visible manifestation of sea level rise and one for which exposed communities are not prepared. The U.S. National Weather Service categorizes tidal flooding that is limited in extent and duration as “minor” or “nuisance” flooding and often issues a Coastal Flood Advisory (CFA) in association with such events. While these floods typically do not pose a direct risk to life or property, they present challenges to daily life in affected areas [17]. Moderate coastal floods today are associated with a Coastal Flood Warning (CFW) about imminent or immediate flooding that could pose a serious risk to life and property.

Both minor and moderate flooding can be detected by higher than normal water level observations at tide gauges maintained by the National Oceanic and Atmospheric Administration (NOAA). The issuance of a CFA or CFW is typically tied to a high tide that is predicted to exceed either the minor or moderate flooding threshold for an area. Based on correlations between water levels at a tide gauge and reported instances of observed flooding, those thresholds are defined observationally by personnel from local National Weather Service offices during and following flooding events—rather than statistically as, for example, the 100-year flood level is.

Several recent analyses of water level observations at NOAA tide gauges have shown that sea level rise is contributing to more frequent and longer-lasting tidal flooding than in decades past [18–21]. Long-term trends show that minor coastal flooding along the East, Gulf, and West Coasts occurred only about once every one to five years in the 1950s, but was occurring about once every three months by 2012 [19]. In several communities, tidal flooding has quadrupled in frequency over the past 40 years [18–20]. This trend is accelerating in many places along U.S. coasts [19]. In communities such as Norfolk, VA, tidal flooding resulting from sea level rise is necessitating changes such as restoring waterfront parks to salt marshes and raising homes [22–24]

In this study, we utilize data from tide gauges maintained by NOAA to determine the frequency of minor tidal flooding events today and for two near-term time horizons that fall within typical infrastructure planning horizons: 2030 and 2045 [25]. This work builds upon previously published projections of tidal flooding frequency in several important ways. First, we apply a screening to the tide gauges used in the analysis to ensure that the observationally-defined flooding thresholds describe the onset of flooding conditions as determined by the issuance of CFAs or CFWs. Second, we utilize localized sea level rise projections based on the Intermediate-Low, Intermediate-High and Highest scenarios from the U.S. National Climate Assessment (NCA; 1,21) rather than the Relative Concentration Pathways (RCPs) defined by the IPCC, as previous studies have done [27,28]. Because the RCP sea level rise projections are produced from process-based models, they are limited by uncertainties in, for example, ice sheet response to warming temperatures and may not capture the upper end of potential sea level rise for this century [29]. In contrast, the NCA Intermediate-High scenario, based on semi-empirical models, projects a higher amount of sea level rise for the middle of this century [26]. Third, we focus on the frequency of flood events rather than on days of flooding or hours of flooding as previous studies have done because many of the adaptations communities have developed to cope with frequent tidal flooding—such as moving vehicles to avoid saltwater exposure—necessitate an awareness of each individual flood event [18,27]. Finally, because the general public often perceives climate change as a temporally distant threat, we have chosen to focus on two time frames (15 and 30 years into the future) that are easily comprehensible within a human lifetime [30–32]. An individual could, for example, view these results through the lens of their 30-year mortgage. Even within these relatively short time frames, we find nearly ubiquitous increases in the frequency of tidal flooding using three different future sea level rise scenarios.

## Methods

### Tide gauge set and flooding thresholds

**Fig 1:**
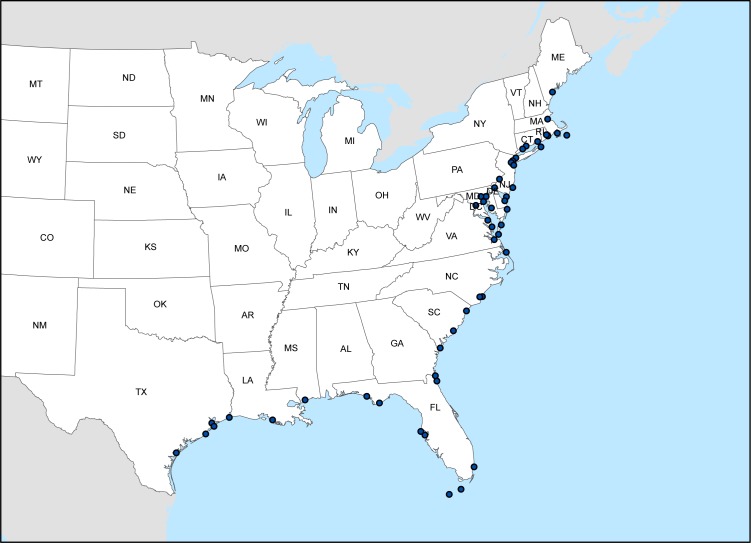
Location of NOAA tide gauges utilized in this study. NOAA’s National Ocean Service maintains all of the 52 tide gauges along the U.S. East and Gulf coasts used in our analysis. We chose gauges with a defined flooding threshold for minor coastal flooding and those included in NOAA’s online Inundation Analysis tool.

A set of 59 tide gauges along the U.S. East and Gulf coasts forms the basis of our analysis, and 52 of those gauges were ultimately determined to have data usable for our purposes (Fig 1). NOAA’s National Ocean Service maintains all of the gauges used in our analysis. We only included gauges that have a defined flooding threshold for minor coastal flooding and are available for analysis within NOAA’s online Inundation Analysis tool [33].

We obtained minor and, where defined, moderate flooding thresholds from the National Weather Service’s Advanced Hydrologic Prediction Service websites (e.g. NWS 2015 [34]). Each threshold was verified by contacting the appropriate Weather Forecast Office. In a few instances where the thresholds could not be confirmed, we relied on thresholds provided by the NOAA Coastal Services Center. We used these observationally based flooding thresholds because they reflect water levels associated with observed local flooding. It is important to note, however, that the flooding threshold for a particular tide gauge may not apply to neighboring communities.

### Flood frequency analysis

To calculate how frequently the minor coastal flooding threshold is currently exceeded at a particular gauge, we have used NOAA’s Inundation Analysis (IA) tool [33]. The IA tool allows the user to set a specific water level—for example the level required to exceed the minor or moderate flooding thresholds—and a specific date range. The tool then analyzes 6-minute water level observations from NOAA tide gauges for the specified date range and returns each event that exceeded the specified flooding threshold as well as basic statistics about each event, such as its elevation above the threshold and its duration. An event is defined as any time the water level exceeded the flooding threshold. By this definition, a location that has two high tides per day could have two flood events per day—each high tide that exceeded the flooding threshold would register as an event. (Instances in which the tidal level briefly but repeatedly surpassed the flooding threshold during a single high tide cycle are not counted as multiple events.) In locations such as Jamaica Bay, NY, and Norfolk, VA, tidal flooding already requires that residents on certain streets move their cars during high tides to avoid salt water exposure. For these sorts of places, the number of events—rather than simply the days with flooding or the hours of flooding, as previous studies have reported—is a direct driver of adaptation actions observed in neighborhoods with frequent tidal flooding.

It is important to note that non-tidal variability due, for example, to wind and storms could raise local water levels above the flooding threshold. While we refer to the flood events as “tidal flooding,” non-tidally driven events also contribute to the flood frequency recorded at a given tide gauge.

To define a current flood frequency, we evaluated three five-year time periods (2001–2005; 2006–2010; and 2011–2015) and, ultimately, used the full the 15-year period as a baseline. Eleven of the 52 studied tide gauges demonstrate a statistically significant increase in the annual number of flooding events over the 15-year period. This increase could reflect anthropogenic sea level rise, natural variability, or a combination of the two.

Previous studies have shown that minor flooding frequency has been increasing over the last 50 years as sea level has risen [19]. The trend for the 2001–2015 period may or may not be consistent with such studies (see section 3.3). Natural variability, however, can also play a significant role in determining tide heights. In particular, the 18.6 year nodal tidal cycle and the 8.85 year cycle of lunar perigee—which manifests as a 4.4 year modulation because of the combination of the 18.6 and 8.85 year cycles—have been shown to affect tidal datums and extreme water levels in the U.S. and elsewhere [19,35–37]. The 18.6 year cycle, which varies spatially in phase and amplitude, tends to have a greater influence on regions dominated by diurnal tides (e.g. the Gulf of Mexico) whereas the 4.4 year cycle tends to have a greater influence on semidiurnal regions, such as the U.S. East Coast [36]. For the sites analyzed here, the amplitude of the 18.6 year modulation is 2 cm or less [38]. Along the U.S. East Coast, the 18.6 year and 4.4 year cycles affect the 99.9^th^ percentile tidal level by 1% and less than 5% of the full tidal range [36]. In the Gulf of Mexico, the 18.6 year and 4.4 year cycles affect the 99.9^th^ percentile tidal level by about 15% and less than 5%, respectively [36].

By using the years 2001–2015 as an evaluation period, our results implicitly incorporate the sea level rise and associated increases in flood frequency that have taken place over the 20^th^ century and provide a baseline frequency that communities are familiar with from direct, recent experience. We then calculated an average flood frequency per year over those 15 years to even out seasonal and inter-annual variability in tide heights from, for example, years with more frequent storms, changes in the Southern Oscillation Index, and extreme sea level rise events such as that that occurred along the Northeast U.S. coast during 2009–2010 [39]. Our 15-year baseline encompasses 80% of the most recent 18.6-year nodal tidal cycle and more than three 4.4 year cycles [40]. Use of this 15-year period therefore minimizes any one phenomenon’s influence on the baseline average. We report the annual average number of events and one standard deviation for the 2001–2015 baseline period.

The tide gauge data have a range uncertainty of +/- 1-2cm in high and low water measurements and a range of +/- 1-5cm uncertainty in the tidal datum elevation mark [33]. While most of the tide gauge records are complete from 2001–2015, 7 of the 52 have one or more calendar years excluded from the analysis due gaps of 5 or more consecutive months (see SOM).

The data derived from the IA tool do not have an associated inherent uncertainty–flooding either occurred or it did not according to the verified records. Because of the implicit uncertainty incorporated into our analysis by evaluating three distinct sea level rise scenarios, the future flood frequency analysis should be considered an indicator of things to come rather than a predictor.

### Confirmation of flood events with coastal flood advisory records

To confirm that exceedances of the flooding threshold are consistently associated with the issuance of CFAs, we examined each flood event identified by the IA method for each tide gauge for the two-year period 2012–2013 and determined whether it was correlated to a specific CFA or other National Weather Service-issued message related to coastal flooding (that is, Coastal Flood Statements, Warnings, and Watches as well as Hurricane and tropical Storm Statements, Advisories, Warnings, and Watches). Gauges with three or fewer flood events during the 2012–2013 period were not assessed, but were included in subsequent analyses. Records of the issuances were evaluated for the county containing the tide gauge and accessed via the Iowa State Mesonet VTEC Browser [41]. We chose 2012–2013 because it was the most recent two-year period at the time of the analysis and because Weather Forecast Offices adjust flooding thresholds over time based on observations.

To ensure that the gauges used in the study provided robust data regarding the existence of local flooding conditions, we only include locations where two-thirds or more of the coastal flooding events identified by the IA have an associated CFA (or other coastal flood statement). Reasons why the IA events and CFAs do not have a 1:1 correspondence are discussed in the Results section. The correspondence between IA flood events and CFAs was used for confirmation of the existence of local flooding rather than as a predictive tool. We did not adjust the flooding thresholds defined by the Weather Forecast Offices based on this analysis. Rather, we used the CFAs to verify the correspondence between flood events defined solely by water level and those defined by local observations. It is important to note that while CFAs are issued at the county level, not all communities within that county will experience tidal flooding given water levels at the flooding threshold.

### Sea level rise projections

We evaluate tidal flooding frequency at two sea level rise futures, 2030 and 2045, using localized versions of three global projections developed for the most recent U.S. National Climate Assessment [26]. Because we use a 15-year period to evaluate current and future flood frequency, it is important to note that our 2030 and 2045 flood frequency calculations represent averages over 15-year periods that center on those years. Local sea level rise projections were developed for each global scenario and provided by researchers at Climate Central [1,26,42,43]. To generate their projections, Climate Central evaluated the historical rate of sea level rise at each gauge and separated out a local component (i.e. the difference between the global average rate and the rate at that gauge). They then added the local component (keeping it steady) to a global sea level rise projection to calculate a gauge-specific sea level rise projection. They have used the Intermediate-Low (IL), Intermediate-High (IH), and Highest (H) National Climate Assessment scenarios and have calculated the amount of sea level rise projected at each gauge for each decade through the end of this century [26,42] (Table 1). This methodology is similar to the group’s earlier work using different global projections [42]. All three projections are included in our analysis.

**Table 1:** Median flood events per year for the Intermediate-High scenario by region.

Region	Current	2030	2045
Northeast	1	5	25
Mid-Atlantic	10	39	169
Southeast	6	29	104
Gulf Coast	0	2	12

Climate Central provided decadal projections for sea level rise through 2100, and we calculated sea level rise for 2045 based on the polynomial fit to the data. Local projections from Climate Central are not available for all of the gauges for which we have flooding thresholds and reliably associated CFAs. In these instances, we have used Climate Central’s nearest available local sea level rise projection to calculate future flood frequency, limiting the allowable distance to that gauge to 100 miles (Table 1).

Sea level rise will affect stretches of our coastline in different ways depending in large part on how land use and coastal morphology change in response to rising water levels. Furthermore, the NCA scenarios underlying the sea level rise projections we use do not have published cones of uncertainty, and we assume that the range of sea level rise projected between the IL and H scenarios is greater than the uncertainty associated with any one scenario. For these reasons, we did not explicitly quantify a resulting uncertainty from the use of each scenario in the context of our analysis but are simply clear in the discussion as to the nature of the assumptions. For a more detailed analysis, refer to [26] and [42].

Because of the influence of the 18.6 and 4.4 year cycles on water levels, some have argued that future sea level rise projections be calculated in 18.6-year intervals [44]. Given the relatively small amplitude of the 18.6 year cycle at our sites, we did not choose to calculate future flood frequency in 18.6 year intervals. We note, however, that the approximately 2 cm amplitude of the 18.6 year cycle could cause our future flooding projections to be either under- or overestimated.

### Analysis of future flooding

As sea level rises due to global warming, it becomes easier to reach the flooding threshold because the base water level increases while the flooding threshold remains constant. In essence, the height of the tide required to cause flooding is lower. To simulate this effect, we subtracted the projected amount of sea level rise for a gauge from the height required to reach the current flooding threshold and used the IA tool for this new threshold for the 2001–2015 time period. A “future flood events” analysis typically returns both (i) the same events as the current flooding analysis–though with a longer duration, and (ii) additional events that did not meet the flooding threshold today, but would if sea level were higher.

We have done these calculations for 2030 and 2045 for all three National Climate Assessment scenarios discussed above though, for the purposes of this publication, we have focused our attention primarily on the IH scenario because it falls in the mid-range of the NCA scenarios. We do, however, give an overview comparison of results for all three scenarios in the Results and Discussion section.

To determine when sea level rise would cause tides that, today, cause only minor floods but would reach as high as a present-day moderate flood, we calculated the difference between the present day minor and moderate flood levels and again used the projections of sea level rise to determine how many years from now sea level would increase by that amount. For example, if there is currently a 15 cm difference between a minor and moderate flood in a particular location, and that location will experience 15 cm of sea level rise in 20 years’ time, then by 2032 (20 years from 2012) what would have been a minor flood today will be a moderate flood instead.

There are inherent assumptions in our analysis, such as that land use and coastal morphology will not change appreciably, and that tidal ranges will continue to be predictable in each location for the short term future (15–30 years), though there is evidence that sea level rise will alter coastal morphology (e.g. [45]) and may also increase tidal range [35,46]. Furthermore, tidal amplitudes and phases can and do exhibit non-stationary changes that could effect future tidal extremes [47,48]. Estimates of non-stationary changes in tidal amplitude vary significantly in both magnitude and direction. At the Boston, MA, tide gauge, for example, the amplitude M2 component increased by 4.3 cm per 100 years between 1921 and 1980 before decreasing abruptly while the amplitude of the S2 component decreased coherently along the U.S. East Coast by roughly 10%—or 0.5 to 2.7 cm) per century [47,49]. The causes of these changes in tidal amplitude are uncertain and may vary from region to region. Changes in water depth, radiative forcing, and the phase of internal tides have been proposed as drivers of changes in the amplitude of the M2 and S2 tides in various locations (see [50] and references therein). Because the direction and amplitude of such changes vary spatially, it is difficult to estimate how non-stationary changes in tidal amplitude might affect our results. We note, however, that a continuation of the coherent decrease in the S2 amplitude could, by 2045, result in a decrease in tidal amplitude on the order of 1 cm, or about 3% of the overall projected sea level rise. Qualitatively, such a decrease would reduce the frequency of future tidal flooding we calculate.

It is important to note that all projections of future climate change are uncertain. We cannot quantify that uncertainty as it applies to the current study given the limits of the published data. However, our results, present a range of future flood conditions given a range of sea level rise projections.

## Results and discussion

### Inundation Analysis/Coastal flood advisory correlation analysis

**Fig 2:**
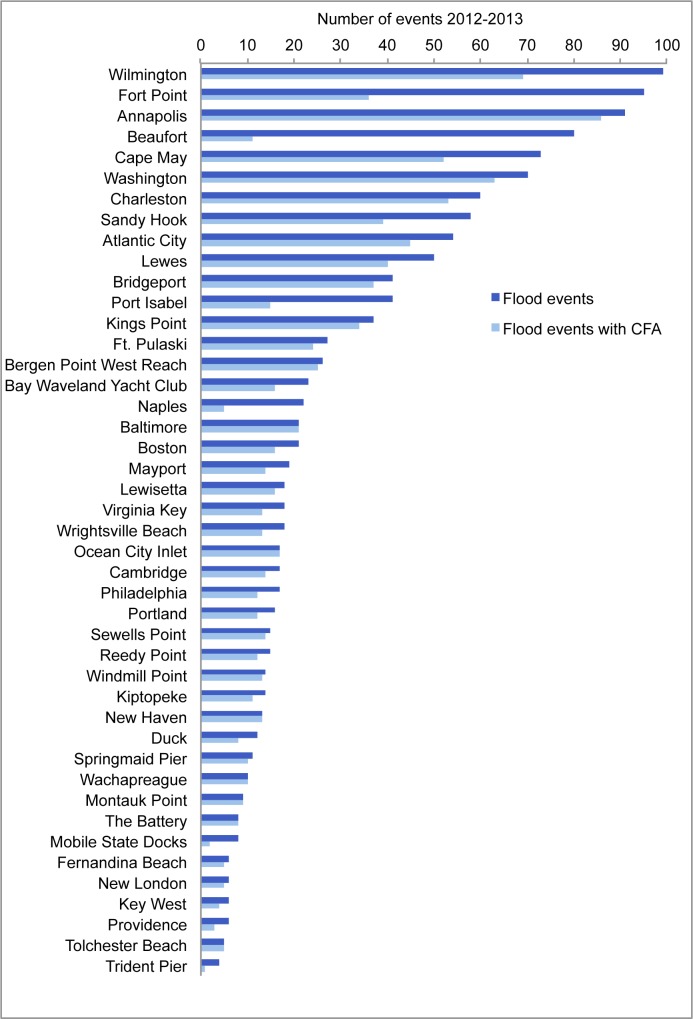
Flood events identified by the Inundation Analysis tool (dark blue) and those with an associated Coastal Flood Advisory (light blue) for the years 2012 and 2013. Gauges where less than two-thirds of flood events identified by the Inundation Analysis tool had corresponding Coastal Flood Advisories were excluded from further analysis. Note that 15 gauges with three or fewer flood events during the 2012–2013 evaluation period were excluded from this correlation analysis.

For 52 of the 59 tide gauges analyzed, two-thirds or more of the flood events identified by the IA were associated with CFAs or other flood statement. For most gauges, the correspondence was 80% or more (Fig 2). That is, for these gauges, a CFA (or other statement) is issued at least 2 out of 3 times when water levels exceed the flooding threshold. Several of the tide gauges had no basis for evaluation because there had been three or fewer coastal flood events occurring during the evaluation period. After ensuring that no CFAs were issued at these gauges during the evaluation period, we included them in the analysis.

Most gauges had instances of CFAs being issued in the absence of an event that exceeded the flooding threshold. There are instances, for example, of Weather Forecast Offices issuing an advisory based on tidal predictions that do not manifest as being above the flooding threshold. Alternatively, strong winds could induce flooding conditions despite water levels falling below the flooding threshold. A large number of issued CFAs without correlative events in the IA would suggest a mismatch between threshold recorded at the tide gauge and the coastal flood advisories as inferred from the number of CFAs. In such instances, our analysis would be a conservative estimate of current and future flood frequency.

There are many potential reasons why exceedances of the flooding threshold might not be associated with a CFA. In some locations, advisories are not issued if the tide is predicted to be just slightly above the flooding threshold for a very short period of time. In less populated regions, the tide may technically exceed the flooding threshold, but the Weather Forecast Office may not issue a CFA. In other locations, such as coastal New Hampshire, the National Ocean Service tide gauge may be located in a different tidal environment than the nearby areas that are most flood-prone, making recorded tidal levels disconnected to the experience of flooding. Wind and wave height can also play into whether or not a CFA is issued, which may affect the correlation between tide height and advisories.

### Sea level rise projections for 2030 and 2045

The IH scenario represents the average of the high end of semi-empirical models that use observed data to extrapolate into the future [51–53]. Under this scenario the 52 gauges utilized in this study are projected to experience an average of 14.6 +/- 2.8 cm of sea level rise by 2030 and 31.5 +/- 5.2 cm by 2045.

**Fig 3:**
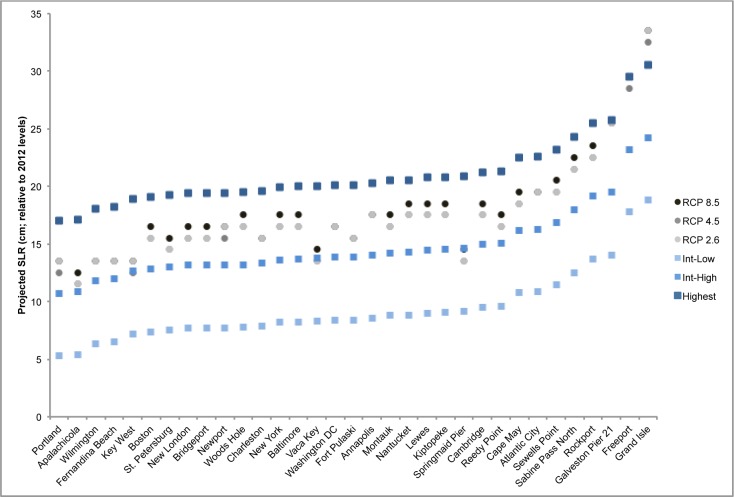
Comparison of local sea level rise projections for 2030. IPCC RCP scenarios (RCP 2.6, RCP 4.5, RCP 8.5) are shown in greyscale circles [28]. National Climate Assessment scenarios (Intermediate-Low, Intermediate-High, and Highest) are shown in bluescale squares ([43]; this study). RCP data represent the 50th percentile projections [28]. Note that this is a subset of our full tide gauge set, as not all gauges included in this study were analyzed by both [28] and [43].

**Fig 4:**
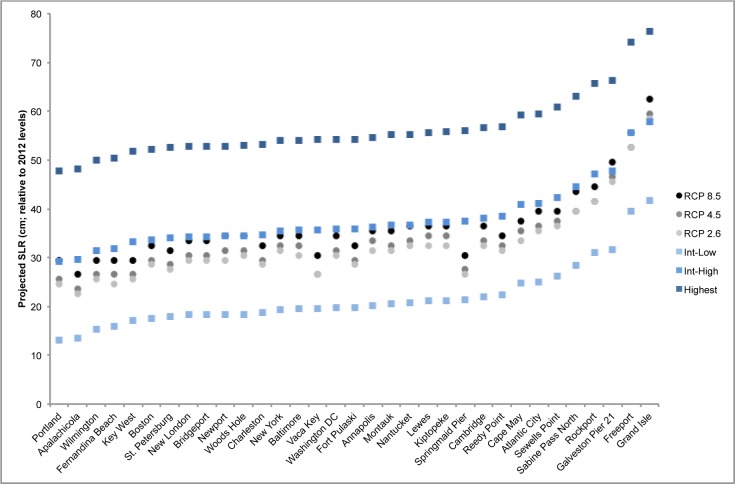
Comparison of local sea level rise projections for 2050. IPCC RCP scenarios (RCP 2.6, RCP 4.5, RCP 8.5) are shown in greyscale circles [28]. National Climate Assessment scenarios (Intermediate-Low, Intermediate-High, and Highest) are shown in bluescale squares ([43]; this study). RCP data represent the 50th percentile projections [28]. Note that this is a subset of our full tide gauge set, as not all gauges included in this study were analyzed by both [28] and [43].

Overall, the localized IH projections agree well with recently published local sea level rise projections using three IPCC scenarios [28,54]. For 2030, the IH projections used here tend to fall slightly below the 50^th^ percentile projections for RCP 2.6, RCP 4.5, and RCP 8.5 (Fig 3). Because data for the RCP projections are not available specifically for the year 2045, we compare the 2050 values for the projections we used with the RCP projections for the same year. For 2050, the IH projections tend to fall slightly above the 50^th^ percentiles of all three RCP projections (Fig 4). The IL and H scenarios generally fall well outside of the full range (5^th^ to 95^th^ percentile) of projections for each RCP scenario, indicating that, of the three scenarios utilized in this study, the IH projection is most closely aligned with the RCP scenarios.

### Evaluation of current flooding

**Fig 5:**
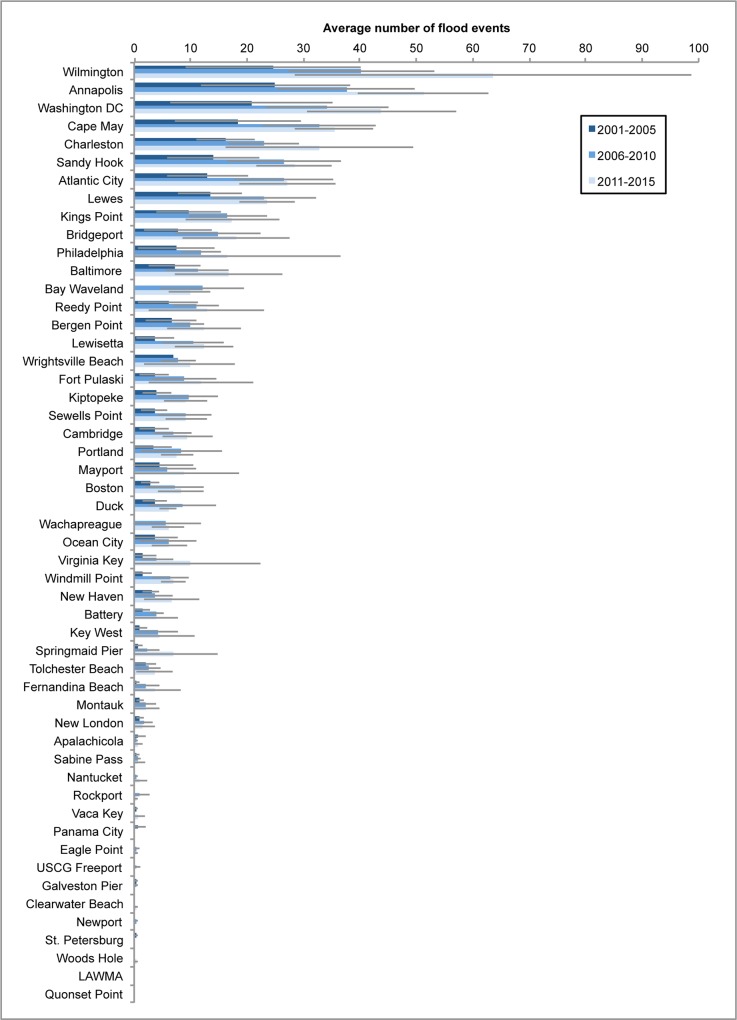
Average tidal flooding events per year for three present-day baseline periods: 2001–2005, 2006–2010, and 2011–2015. Blue bars show averages for each 5-year period, grey bars show one standard deviation.

We evaluated the average number of flood events per year for three distinct baseline periods: 2001–2005, 2006–2010, and 2011–2015 (Fig 5). Between 2001–2005 and 2006–2010, 5 of the 52 studied gauges showed statistically significant (p < 0.05) increases in flood frequency. Between 2001–2005 and 2011–2015, 11 gauges showed such an increase. Using a p-value of 0.01, however, only one gauge shows a statistically significant increase in flood frequency between 2001–2005 and 2011–2015. These comparisons suggest that the choice of a baseline period for present and future flood frequency evaluation could be meaningful. In order to minimize seasonal, interannual, and longer-term variability in our tidal flooding frequency calculations, we used the full 2001–2015 period as a baseline. Results reported in the following sections and Figs reflect the full 15-year baseline period.

### Tidal flooding in 2030 (Intermediate-High scenario)

Five of the 52 locations we analyzed already experience an average of 24 or more tidal flood events per year. This alone is a significant change over historical levels: In 1950, for example, the return period for minor flooding was approximately 1–5 years whereas in 2012, the return period was typically just 3 months throughout much of the United States [19]. For a complete analysis comparing past and present minor flood frequency, please see [19].

**Fig 6:**
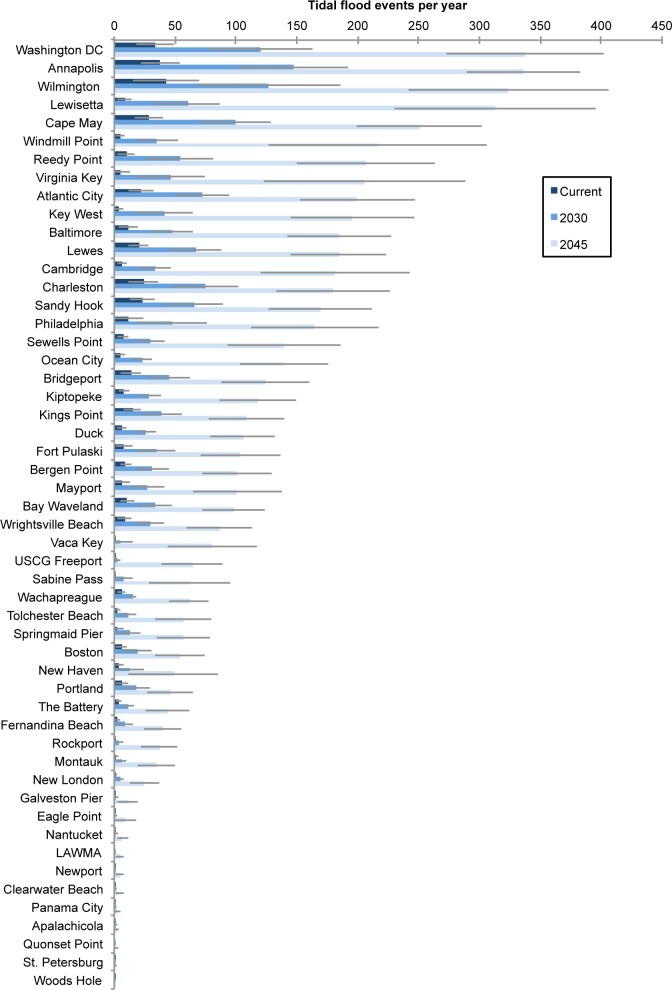
Tidal flooding events per year for all gauges observed today, and projected for 2030, and for 2045. Numbers shown represent annual averages and are based on localized sea level rise projections using the National Climate Assessment’s Intermediate-High scenario.

By 2030, given projected sea level rise in the IH scenario, our projections show that 26 of these communities—half of those analyzed—can expect to face tidal flooding at least 24 times annually (Fig 6). The increase in flooding is statistically significant (p < 0.01) at 38 of our 52 sites and represents a steep increase in flooding for many of these places. Twenty-four of those 26 communities can expect to see the yearly number of high-tide floods triple in just 15 years. Ten of those communities already face frequent tidal flooding and a tripling or more is projected to cause an average of 52 or more floods annually or the equivalent of one flood per week, on average.

**Fig 7:**
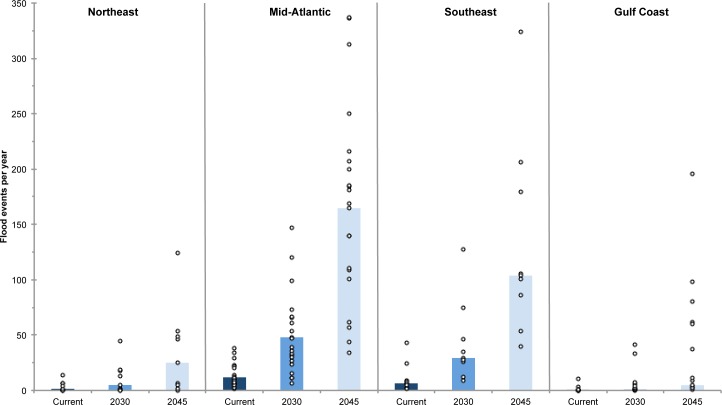
Flood events per year by region. Regions are defined as follows. Northeast: ME, MA, RI, CT; Mid-Atlantic: PA, DE, NJ, NY, VA, MD; Southeast: NC, SC, GA, FL (Atlantic Coast); Gulf Coast: FL (Gulf Coast and Keys), LA, MS, TX. Bars show median values for each region. Open circles show values for each tide gauge within the region.

On a regional basis, the Northeast and Gulf Coast see small increases in the median annual number of flood events by 2030 in the IH scenario, whereas the Mid-Atlantic and Southeast coasts see much larger increases (Fig 7, Table 1). However, that there is considerable variability between gauges within a region, and, as is clear from the range in the number flood events for each tide gauge, regional medians can mask the statistically significant changes at any one gauge. The greatest increases in flooding frequency by 2030 will occur along the Mid-Atlantic coast. By 2030, with this scenario, several New Jersey locations can expect to average 70 to 100 tidal floods a year. And places such as Annapolis, MD, and Washington, DC, can expect to average 120 to 150 tidal floods each year.

**Fig 8:**
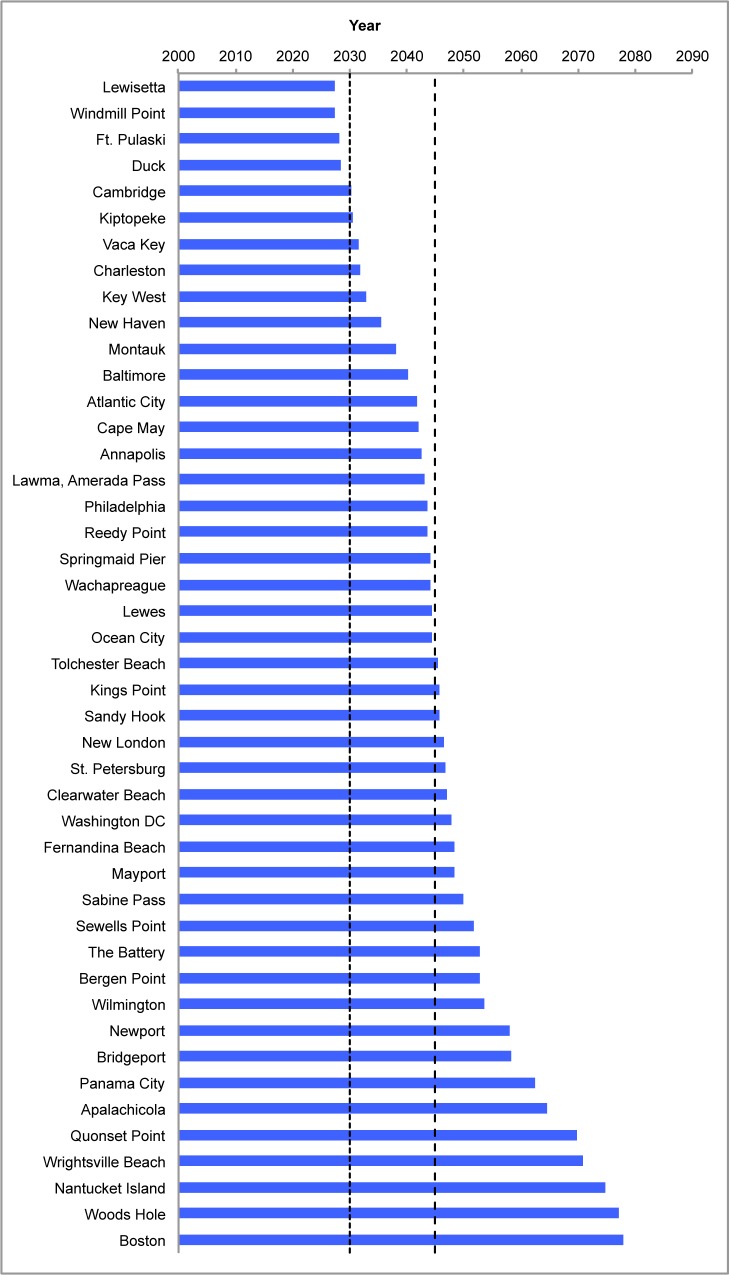
When sea level rise turns a minor flood into a moderate flood. Bars show the year in which a tide that would cause only a minor flood today would cause a moderate flood based on localized sea level rise projections using the Intermediate-High scenario. The two vertical lines are at 2030 and 2045, our two time horizons for analysis.

By 2030, tides that cause only minor flooding today are expected to cause moderate flooding in some locations (Fig 8). At Lewisetta, VA, for example, only about 15 cm currently separate a tide that causes minor flooding from one that causes moderate flooding. That means tides alone could cause moderate flooding when sea level rises by just 15 cm—as expected within 15 years—threatening nearby historic and tourism destinations such as Reedville, VA. Seven other communities among our 52—Cambridge, MD; Charleston, SC; Duck, NC; Kiptopeke, VA; Savannah, GA (at Fort Pulaski); Vaca Key, FL; and Windmill Point, VA—are also expected to face moderate flooding from tides alone by about 2030, because of sea level rise (Fig 8). In places such as Charleston, where residents are already familiar with frequent minor coastal flooding and the occasional moderate flood during heavy rains and storms, less than 15cm of sea level rise would mean that high tides alone could flood substantial areas up to two dozen times per year, on average, by about 2030, assuming no adaptive action.

### Tidal flooding in 2045 (Intermediate-High scenario)

Many coastal communities can expect about a 30 cm increase in sea level by 2045 with the IH scenario, and with that increase, many can also expect highly regular tidal flooding (Table 2). Nearly all sites experience a statistically significant (p < 0.01) increase in flood frequency compared to the present (49 of 52 sites) and compared to our 2030 projections (47 of 52 sites). On a regional basis, these changes are most noteworthy for the Mid-Atlantic region, but all regions experience increases in median flood frequency (Fig 7). Arriving on ever-higher seas, these tides will be able to reach farther into communities, creating flood conditions that can last longer and disrupt life for growing numbers of people [55]. By 2045, within the lifetime of a typical home mortgage, more than half of our 52 communities could experience, on average, a 10-fold or greater increase in the frequency of tidal floods with this scenario. And nearly one-third of the 52 could average more than 180 tidal floods per year (Fig 6).

**Table 2:** Current and future average annual flood frequency at all sites for the Intermediate-High scenario. Also shown are one standard deviation of the average for each time horizon and sea level rise projections for each site [43].

Tide Gauge	Gauge #	2001–2015: events/yr	2001–2015: 1σ	2030: SLR (cm)	2030: events/yr	2030: 1σ	2045: SLR (cm)	2045: events/yr	2045: 1σ	Nearest projection
Annapolis	8575512	37.9	15.9	14.0	147.2	44.9	30.5	336.5	46.7	
Apalachicola	8728690	0.5	0.9	10.9	0.9	1.4	24.7	2.2	2.1	
Atlantic City	8534720	22.3	10.2	16.3	72.7	22.1	34.7	199.7	47.3	
Baltimore	8574680	11.7	7.6	13.7	47.5	17.6	30.0	185.1	42.7	
Bay Waveland YC	8747437	10.9	5.7	11.9	33.1	14.1	26.6	98.2	25.5	Pensacola
Bergen Point	8519483	9.6	5.1	13.6	31.4	13.5	29.8	100.9	28.4	The Battery
Boston	8443970	6.1	4.4	12.8	18.8	11.9	28.3	53.8	20.5	
Bridgeport	8467150	13.5	8.5	13.0	44.7	17.5	28.7	123.9	36.4	
Cambridge	8571892	6.6	4.1	14.9	33.5	13.6	32.2	181.5	61.2	
Cape May	8536110	28.9	11.8	16.2	99.0	29.8	34.5	250.5	51.5	
Charleston	8665530	24.0	12.2	13.3	74.6	27.3	29.2	179.4	46.9	
Clearwater Beach	8726724	0.1	0.3	12.8	0.8	1.3	28.2	4.4	3.3	
Duck	8651370	6.1	4.1	16.9	25.8	8.6	35.8	105.6	26.8	Sewells Point
Eagle Point	8771013	0.2	0.4	19.5	1.1	1.3	40.5	9.2	8.8	Galv. Pier 21
Fernandina Beach	8720030	1.9	2.9	12.0	9.2	6.5	26.8	40.1	15.0	
Ft. Pulaski	8670870	8.1	7.0	13.8	34.6	15.7	30.2	103.7	32.9	
Galveston Pier 21	8771450	0.1	0.4	19.5	1.6	1.8	40.5	11.1	8.8	
Key West	8724580	3.3	4.2	12.6	41.4	23.3	27.9	195.6	51.0	
Kings Point	8516945	14.9	7.5	13.6	39.0	16.7	29.8	108.9	31.0	The Battery
Kiptopeke	8632200	7.9	4.6	14.6	28.6	9.8	31.5	117.9	31.6	
Lawma, Amerada Pass	8764227	0.0	0.0	24.3	0.6	1.1	49.3	4.8	3.0	Grand Isle
Lewes	8557380	20.0	8.0	14.5	66.8	21.1	31.4	184.4	39.0	
Lewisetta	8635750	8.9	5.8	18.0	60.5	26.7	37.7	312.6	82.8	
Mayport	8720218	6.4	7.0	12.0	27.4	13.7	26.8	100.9	36.8	Fernandina Bch.
Montauk	8510560	1.7	1.8	14.2	6.1	3.7	31.0	34.5	15.5	
Nantucket Island	8449130	0.4	0.9	14.3	1.6	1.7	31.0	6.9	4.9	
New Haven	8465705	4.4	3.6	13.0	12.7	12.5	28.7	49.1	36.9	Bridgeport
New London	8461490	1.5	1.5	13.1	4.9	2.8	28.9	25.0	12.1	
Newport	8452660	0.1	0.3	13.1	0.7	1.0	28.9	4.6	3.7	
Ocean City	8570283	5.5	4.0	14.5	23.2	7.7	31.4	139.3	36.5	Lewes
Panama City	8729108	0.3	0.8	10.9	0.7	1.2	24.7	2.7	2.5	Apalachicola
Philadelphia	8545240	11.9	12.1	15.1	48.0	28.5	32.4	164.7	52.8	Reedy Point
Portland	8418150	6.5	5.0	10.7	17.7	11.5	24.4	46.1	19.0	
Quonset Point	8454049	0.0	0.0	12.3	0.1	0.4	27.4	1.5	1.8	Newport
Reedy Point	8551910	9.9	7.2	15.1	53.7	27.9	32.4	206.7	56.7	
Rockport	8774770	0.4	1.1	19.2	3.7	3.7	40.0	37.0	15.0	
Sabine Pass	8770570	0.5	0.8	18.0	7.4	7.7	37.7	61.6	33.6	
Sandy Hook	8531680	23.0	10.3	13.6	66.1	23.4	29.8	169.2	42.5	The Battery
Sewells Point	8638610	7.3	4.4	16.9	30.2	11.8	35.8	139.5	46.4	
Springmaid Pier	8661070	3.0	4.8	14.6	12.7	8.8	31.6	56.6	22.1	
St. Petersburg	8726520	0.1	0.3	13.0	0.2	0.8	28.6	0.9	1.2	
The Battery	8518750	3.3	2.6	13.6	11.1	5.3	29.8	43.6	18.1	
Tolchester Beach	8573364	2.7	2.4	13.7	11.0	7.4	30.0	56.9	22.8	Baltimore
USCG Freeport	8772447	0.1	0.4	23.2	2.3	3.0	47.4	64.0	24.9	Freeport
Vaca Key	8723970	0.3	0.7	13.8	5.7	9.5	30.0	80.5	36.6	
Virginia Key	8723214	5.1	7.9	13.8	46.0	28.5	30.0	205.9	82.8	Vaca Key
Wachapreague	8631044	5.9	3.5	14.6	15.3	3.3	31.5	61.6	15.8	Kiptopeke
Washington, DC	8594900	33.8	15.2	13.8	120.2	43.0	30.2	337.2	64.8	
Wilmington	8658120	42.8	27.3	11.8	127.3	58.5	26.4	323.7	82.5	
Windmill Point	8636580	5.0	3.4	18.0	35.3	17.1	37.7	216.2	89.8	Lewisetta
Woods Hole	8447930	0.1	0.3	13.2	0.1	0.3	29.0	0.2	0.6	
Wrightsville Beach	8658163	8.6	5.6	11.8	29.3	11.6	26.4	86.2	27.0	Wilmington

Vulnerable coastal land areas may not be permanently inundated—that is, submerged during all high tides—until late this century or beyond. However, eight of the 52 locations we studied would face an average of 200 or more tidal floods a year with the IH scenario. That means some areas in those locations could be effectively inundated in just the next few decades, by virtue of being so often underwater (Fig 6).

For some of these locations, the changes in flood frequency in the next three decades could be steep. In New London, CT, for example, even minor tidal floods now occur just twice per year, on average. By 2045, however, high tides could bring 25 ± 12 tidal floods to the city every year. Other locations that have fewer than five tidal floods per year today could see a 10-fold or greater increase in the frequency of floods by 2045. Along the Gulf Coast, in particular, can many new areas would be exposed to tidal flooding with this scenario. Several Gulf Coast locations currently experience little to no tidal flooding, including Freeport, Rockport, and Sabine Pass, TX, could face 35 to 60 tidal floods per year by 2045.

As sea level rises, tidal floods will reach farther inland and expose new areas to flooding. By 2045, nearly half of our 52 communities can expect normal tidal fluctuations to bring moderate flooding (Fig 8). The coastal areas of Delaware, Long Island, and Maryland, for example, will face moderate flooding from tides alone. The combination of more frequent and more severe floods will affect many locations, including Philadelphia, PA, which is expected to see moderate tidal flooding an average of 12 times per year by 2045.

**Fig 9:**
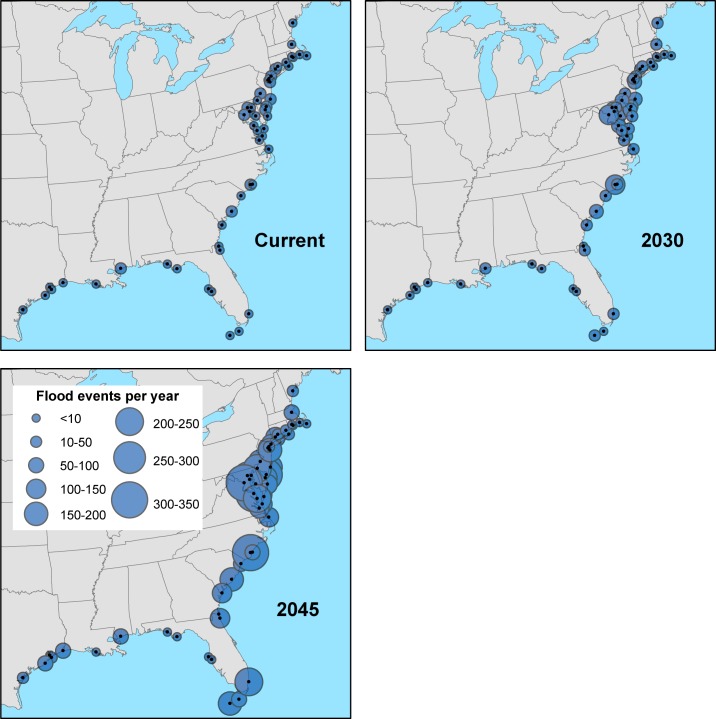
**Average annual number of flood events today (A), in 2030 (B), and in 2045 (C).** Numbers are based on localized sea level projections using the National Climate Assessment’s Intermediate-High scenario. Circle size represents the average number of flood events per year.

While these results apply specifically to the tide gauges we analyzed and not neighboring communities, the nearly ubiquitous rise in flood frequency under the IH scenario in the 52 locations studied suggests that communities not covered by our analysis can expect to see changes on a similar scale, depending on their local topography, natural and physical defenses, measures to accommodate the changes, and other preparatory steps (Fig 9).

### Results from the Intermediate-Low and highest scenarios

The Intermediate-Low (IL) scenario assumes aggressive decreases in GHG emissions consistent with the IPCC AR4 B1 scenario [26]. In this projection, sea level rise is primarily driven by thermal expansion, with minimal ice loss. The Highest (H) scenario, in contrast, assumes a maximum level of loss of land ice and ocean warming consistent with the IPCC AR4 A1B scenario [26,56]. As noted above, the IL (H) scenario projects notably less (more) sea level rise for the gauges in this study than the full ranges (5^th^ to 95^th^ percentile) of the RCP 2.6, 4.5 and RCP 8.5 scenarios [28]. The three scenarios utilized in this study project significantly different amounts of sea level rise (Table 3), and our results for future flood frequency and extent track those differences accordingly (S1 Table).

**Table 3:** Average local sea level rise (in cm) at the gauges utilized in this study.

Scenario	SLR: 2030 (cm)	SLR: 2045 (cm)
Intermediate-Low	9.1 ± 2.8	18.1 ± 5.2
Intermediate-High	14.6 ± 2.8	31.5 ± 5.2
Highest	20.9 ± 2.8	45.5 ± 5.2

**Fig 10:**
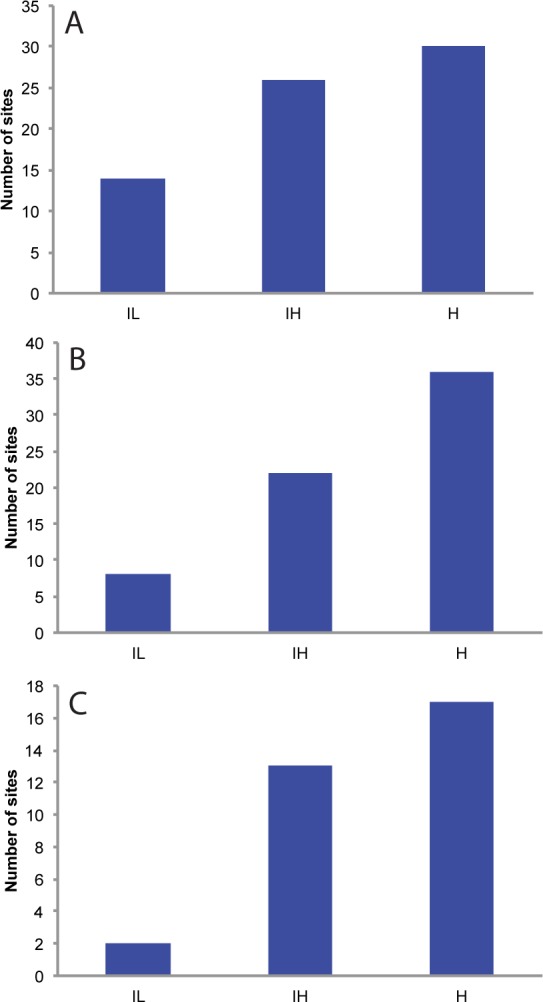
**A) Number of sites with, on average, 24 or more minor coastal flooding events per year in 2030**. **B) Number of sites at which a flooding event that would cause only minor flooding today would cause moderate flooding in 2045. C) Number of “newly exposed” sites. Number of sites with fewer than 5 minor flooding events per year today that have at least 24 flooding events per year in 2045 and a 10-fold increase in the number of flood events.** Numbers are based on localized projections for the Intermediate-Low (IL), Intermediate-High (IH) and Highest (H) scenarios.

The number of sites experiencing 24 or more flood events per year increases progressively from the IL to the H scenario. Today, only 5 sites experience 24 or more floods events per year. By 2030, 14, 26, or 30 sites are projected to experience this frequency of flooding for the IL, IH, and H scenarios, respectively (Fig 10A). From the IL to the IH scenario, the average number of flood events roughly doubles. The average number of flood events increases nearly 3-fold when comparing the IH and H scenarios.

Similarly, the extent of flooding increases linearly as the pace of sea level rise increases such that by 2045, sites at which the tides that cause only minor flooding today would be causing moderate flooding in 8, 22, or 36 sites, respectively, for the IL, IH, and H scenarios (Fig 10B).

The number of sites that will join the ranks of the frequently flooded in the future increases significantly with increasingly severe sea level rise. By 2045 2/13/17 (IL/IH/H) sites that currently experience fewer than 5 flood events annually could experience more than 24 flood events and a 10x increase in flood frequency (Fig 10C).

### Comparison to other tidal flooding studies

Our results agree qualitatively with those of Sweet and Park (2014) who employed localized sea level rise projections based on RCP projections to evaluate the exceedance probabilities for an overlapping set of gauges [27,28]. Sweet and Park (2014) found that, regardless of the RCP projection, the majority of sites they evaluated had 30+ days of flooding per year within the next few decades. Similarly, we find that 79% of our gauges cross that level of flooding by 2045. Differences between our results from Sweet and Park (2014) may arise from the fact that their study uses a metric of “days with flooding” whereas we utilize “flooding events”. Because most places along the East Coast have two high tides per day, a day with two high tide floods would count as two events in our study, but only one in Sweet and Park (2014).

Sweet and Park (2014) present data for the annual number of flood days based on local flooding thresholds for only one site included in our analysis: Norfolk/Sewells Point, VA. At this site, the projected number of flood days based on all three RCP projections falls between our projections for the Intermediate-High and Intermediate-Low projections. Whether this difference—and, potentially, differences at other sites—results from the different sea level rise projections or different methodologies could be a subject for future investigation.

## Conclusion

When tidal floods occur, water can cover coastal roads for hours, making passage risky or impossible [57]. With water on the street, some residents can be effectively trapped in their homes, and homes can be damaged [58]. Entire neighborhoods can be affected, even isolated [59]. In many communities, retail stores, restaurants, other businesses, and public infrastructure are clustered in low-lying waterfront areas, in easy reach of tidal flooding [60].

In decades past, high tide flooding had little impact on coastal communities because our shorelines were not as heavily developed and sea level was not as high. Today, however, the reach and effect of the tides is changing, and many coastal towns and cities are already grappling with how best to protect their communities and infrastructure.

Based on localized sea level projections for 2030 and 2045, we project a nearly ubiquitous increase in the frequency and extent of tidal flooding along the U.S. East and Gulf coasts. Coastal communities and states, and the nation as a whole, need to prepare for near-term changes in tidal flooding, while working hard to minimize longer-term losses through efforts to both adapt to these changes and limit their extent. This preparation should include federal efforts to sustain and expand the nation’s tide gauge network to ensure that local decision makers have access to the best possible data.

## Supporting information

S1 Table Full study dataset. Tidal flooding events per year for all tide gauge locations and sea level rise scenarios evaluated in this study.
(XLSX)
